# Cases Extracted, by Permission, from the Note-Books of Henry Davies, M.D.

**Published:** 1833-10

**Authors:** 


### ORIGINAL COMMUNICATIONS.

*Cases extracted, by permission, from the Note-books of* HENRY DAVIES, M.D., Physician-Accoucheur to the Brownlow-street Lying-in Hospital, &c.

CASE I. Ruth Parberry, aged forty-three, the mother of five children, applied for advice at the Welbeck-street Dispensary, July 7, 1828. Between two and three years before this period, she first perceived a tumour in the lower part of the belly, occupying the situation of the uterus, and of the size of that organ about the third month of pregnancy; it was not painful, nor did it cause her any inconvenience. Some months after its appearance she became subject to attacks of menorrhagia, attended with violent bearing-down pains, the blood being generally discharged in large coagula. These attacks continued at irregular intervals till she fell pregnant, fourteen months ago, and did not entirely cease till the period of quickening. During the latter months of pregnancy, she suffered much from excruciating pains in the abdomen, which came on in paroxysms, and were not confined to any one part. The labour, she states, was tedious and severe. Since the birth of the child the pains have subsided, and there has been no return of menorrhagia; within the last two or three months small substances resembling pieces of skin have been discharged from the vagina, but there has been no leucorrhœa. The tumour has gradually increased in size, and now occupies nearly the whole of the hypogastrium. She often suffers from nausea, and a sensation of sinking at the pit of the stomach. Much exercise produces dyspnœa, and there is a constant sense of weight and numbness in the lower extremities, with a dull pain in the loins. She has no other symptoms of disease; the urine is retained and voided without difficulty; the digestive functions are unimpaired, the circulation natural, and her general appearance denotes perfect health. Among the various remedies which have been employed she has found most relief from the local abstraction of blood, but this has produced only a slight and temporary diminution in the size of the tumour. Liniment. Hydrarg. hypogastrio infricetur. Sumat Tr. Iodin. gtt. x. quotidie.

July 28. Sumat Tr. Iodin. gtt. xij. quotidie.

August 18. Sumat Tr. Iodin. gtt. xxv. quotidie; Rep. Liniment. Hydrarg.

September 1. The hypogastrium is two inches less in circumference. Sumat Tr. Iodin. gtt. xv. ter de die; Applicentur Hirud. hypogastrio.

15. The tumour is larger, and the patient complains of headach. Omit. Tr. Iod. Sumat Haust. cum Rhei pulv. et Magn. Applicentur Hirud. xij. hypogastrio.

29. The Catamenia returned last week, though she still continues to suckle. She feels better in every respect, and the tumour is evidently smaller. Resumatur usus Tr. Iodin.

June, 1829. The patient is extremely weak and emaciated, after a recovery from an attack of inflammation of the bowels. The tumour, on measuring her, is found to be larger.

October. A couple of months since, she was suddenly seized with severe bearing-down pain, and profuse floodings, which weakened her so much that she rallied with difficulty. Since this attack her health has given way, and she has rarely been free from pain in the abdomen. The tumour is larger, and distends the whole cavity.

December. The abdomen is immensely distended; the tumour is felt more distinctly on the right side; the integuments are tense, and there is an evident fluctuation. The patient complains of severe lancinating pains in different parts of the abdomen, but particularly on the right side; the tumour rises to the right hypocondrium, where there is a considerable degree of soreness, and the patient cannot lie on the opposite side, from the sense of dragging she feels at this part. She is emaciated, and confined to her bed.

January, 1830. The abdomen daily becomes more tense. There is general soreness on pressure, and acute lancinating pains in different parts of the tumour, and also in the loins. At the latter end of the month she was admitted into the Westminster Hospital, where she remained for a few days, and was then sent home. Shortly after her return the integuments gave way at the umbilicus, and, according to her account, a thick discharge of a red colour issued from the orifice, and gave her great relief.

July 30, 1833. Mrs. Parberry has generally enjoyed tolerable health since the last report. She occasionally suffers from violent pain and tension of the abdomen, but this is eventually relieved by a discharge from the umbilical orifice, which admits a probe easily. She was this day found actively engaged in her avocations as a laundress.

Case ii. January 27, 1819. Sarah Larman, æt. forty-three, has been a widow these twelve years. She has enjoyed very good health until eighteen months ago, when, after straining herself by lifting a heavy weight, she was immediately seized with a violent flooding, which continued for eight months; this was succeeded by a tumour in her left side, which gives her great pain, and her abdomen has since gradually enlarged. She has been in St. George's Hospital, where she was cupped, salivated, &c. There is perceptible fluctuation more particularly on the right side; her bowels are irregular, and she does not make water oftener than once a day. She sleeps ill; her countenance is pale, and her tongue exsanguine; her pulse eighty and of moderate fulness. ℞. Pil. Hydrarg. gr. iv.; Opii pulv. grss. ft. Pil. o. n. sumenda. Sumat Potassæ Supertart. ʒi. bis in die.

February 7. Cont. Med. sed adde pil. Extr. Elater. gr. ss.

10. Has been much purged. There is retention of urine, and acute pain over the hypogastrium; pulse seventy-five. Hirud vi. loco dolenti. Postea appl. Empl. Canth. Sumat Mist. Salin. cum Pulv. Ipecac. C.

15. Is much better.

March 20. She is obliged to have her urine drawn off; and, with the exception of two days, has passed it voluntarily but twice. The abdomen is tender on pressure. ℞. Potassæ Supertart. ℥ss.; Sp. Juniperi ℥ij.; Aq. Puræ ℥xiv. M. sumat cyath. vinos. bis in die. ℞. Hydr. Subm. gr. i.; Ext. Opii gr. i.; Ext. Hyoscyam. gr. ij. M. fiat pil. h. s. s. Lin. Ammon. c̄ Hydr. abdom. o. n. infricetur. Let her water be drawn off twice a day.

April 3. Her general health has improved, but the mercurial was discontinued on account of the soreness of her mouth. Cont. Mist. Sumat Extr. Hyoscyami ℈ss. h. s. Let dry friction of the abdomen be employed, and let the water be drawn off twice a day.

July 1. Her health has improved, and the pain from distension is much less. She was now furnished with a catheter. Her abdomen became enormously distended, and she went on with variable health till

August, 1825, when she had a severe attack of abdominal inflammation, which was treated with copious venesection and cupping; she was also unintentionally salivated by the exhibition of calomel and opium. A profuse serous discharge from the vagina now followed, so as to require four or five napkins daily; and the abdomen gradually diminished in size until it became comparatively flat towards the beginning of October. From this time she enjoyed tolerable health, and was much employed as a nurse, until her death, by an apoplectic attack, in the spring of 1830. A post-mortem examination was obtained, and the right lobe of the liver was found to be entirely attached to the peritoneal lining of the lower ribs. The uterus was firm in its structure, and attached to the peritoneal covering of its fundus; a fleshy membranous tumour of small size, having no very distinct or decided character, was adhering to it. This was apparently the remains of the ovaria and fallopian tubes, but there was no cyst.

